# Metabolic and Proteomic Analysis of *Chlorella sorokiniana*, *Chloroidium saccharofilum*, and *Chlorella vulgaris* Cells Cultured in Autotrophic, Photoheterotrophic, and Mixotrophic Cultivation Modes

**DOI:** 10.3390/molecules27154817

**Published:** 2022-07-27

**Authors:** Agata Piasecka, Andrea Baier

**Affiliations:** 1 Department of Physical Properties of Plant Materials, Institute of Agrophysics, Polish Academy of Sciences, Doswiadczalna 4, 20-290 Lublin, Poland; 2 Department of Animal Physiology and Toxicology, The John Paul II Catholic University of Lublin, Konstantynow 1 I, 20-708 Lublin, Poland; andrea.baier@kul.pl

**Keywords:** proteins, microalgae, *Chlorella*, proteomics, gel electrophoresis, mixotrophy, beet molasses

## Abstract

*Chlorella* is one of the most well-known microalgal genera, currently comprising approximately a hundred species of single-celled green algae according to the AlgaeBase. Strains of the genus *Chlorella* have the ability to metabolize both inorganic and organic carbon sources in various trophic modes and synthesize valuable metabolites that are widely used in many industries. The aim of this work was to investigate the impact of three trophic modes on the growth parameters, productivities of individual cell components, and biochemical composition of *Chlorella sorokiniana, Chloroidium saccharofilum,* and *Chlorella vulgaris* cells with special consideration of protein profiles detected by SDS-PAGE gel electrophoresis and two-dimensional gel electrophoresis with MALDI-TOF/TOF MS. Mixotrophic conditions with the use of an agro-industrial by-product stimulated the growth of all *Chlorella* species, which was confirmed by the highest specific growth rates and the shortest biomass doubling times. The mixotrophic cultivation of all *Chlorella* species yielded a high amount of protein-rich biomass with reduced contents of chlorophyll a, chlorophyll b, carotenoids, and carbohydrates. Additionally, this work provides the first information about the proteome of *Chloroidium saccharofilum*, *Chlorella sorokiniana*, and *Chlorella vulgaris* cells cultured in molasses supplementation conditions. The proteomic analysis of the three *Chlorella* species growing photoheterotrophically and mixotrophically showed increased accumulation of proteins involved in the cell energy metabolism and carbon uptake, photosynthesis process, and protein synthesis, as well as proteins involved in intracellular movements and chaperone proteins.

## 1. Introduction

Given the climate change, the growth of the world’s population, and the changing diets, food security and sustainable preservation of water and land resources are becoming a key challenge. Satisfying the nutritional needs of the human population requires searching for unconventional sources of nutrients, including protein. The increasing demand for protein is an opportunity for the market of plant-based protein ingredients to expand considerably [1]. Microalgae are one of many promising alternative plants for protein production [2]. Crude protein in microalgal cells makes up from 30 to 80% of dry weight in optimum environmental conditions and contains all essential amino acids needed in diets; hence, it is a complete protein source [3,4,5,6]. *Chlorella* proteins are especially recognized as a product with potential health benefits and are regarded as safe for consumption [7,8]. *Chlorella* species are nutritionally the most important protein-rich microalgae that can be a good natural source of potentially bioactive peptides [6]. Furthermore, microalgae have great potential as a dietary component due to not only the content of high-value protein but also the high energy value and the content of lipids, minerals, and vitamins [9].

Strains of the *Chlorella* genus have been reported to have the capacity to adjust to a variety of environmental conditions and various carbon forms, as they are able to grow autotrophically, heterotrophically, photoheterotrophically, and mixotrophically [5,10]. The most preferable microalgal trophic mode is mixotrophic cultivation [11]. Microalgal cells can utilize organic carbon sources (OCSs) and CO_2_ simultaneously, which contributes to a very rapid increase in cell biomass [12]. The mixotrophic mode based on the use of residues from agro-industry as a source of organic carbon seems to be the most beneficial solution in terms of economy and environment. Agro-industrial wastes, residues, and by-products are of great interest due to their low cost, renewable nature, and abundance [13]. In the post-fossil era, mixotrophy based on sugars and capture of CO_2_ from air is mandatory in microalgal cultivation [3].

In the mixotrophic mode with the use of beet molasses, which is an additional source of mainly carbon and nitrogen, microalgal cells regulate the course of metabolic processes, which results in the synthesis and accumulation of specific proteins [5]. The control of metabolism and targeting the metabolic pathways towards the synthesis and accumulation of specific proteins can be an opportunity for development of many branches of industry, e.g., pharmacy, medicine, and cosmetics. Therefore, it is important to elucidate the processes and mechanisms regulating carbon and nitrogen metabolism in different *Chlorella* species. By combining two-dimensional gel electrophoresis with MALDI-TOF/TOF spectrometry, it is possible to analyze the protein profiles in *Chlorella* cells growing in autotrophic, photoheterotrophic, and mixotrophic cultivation modes. Proteomics techniques provide information necessary for elucidation of the biochemical processes at the molecular level [14]. The proteome is constantly undergoing changes in response to environmental conditions; therefore, results of proteomic analyses provide information on the level of expression of *Chloroidium saccharophilum*, *Chlorella sorokiniana*, and *Chlorella vulgaris* proteins in different trophic modes. The application of proteomic analysis in investigations of the metabolism in the unicellular *Chlorella* genus is an increasingly popular method; however, in a majority of cases, it is confined to elucidation of the mechanisms of regulation of lipid biosynthesis due to the great interest in algal culture for biofuel production [15]. The proteome of *C. saccharophilum*, *C. sorokiniana*, and *C. vulgaris* cells cultured in molasses supplementation conditions has not been identified so far.

To the best of our knowledge, this research is the first comprehensive metabolic and proteomic analysis of three *Chlorella* species: *Chlorella sorokiniana*, *Chlorella vulgaris,* and yet unexplored *Chloroidium* *saccharophilum*. In the case of these species, the mechanisms of protein synthesis and accumulation are not well known, in particular in the conditions of molasses supplementation.

## 2. Results

### 2.1. Effect of the Cultivation Mode on Growth Characteristics

**Figure 1:**
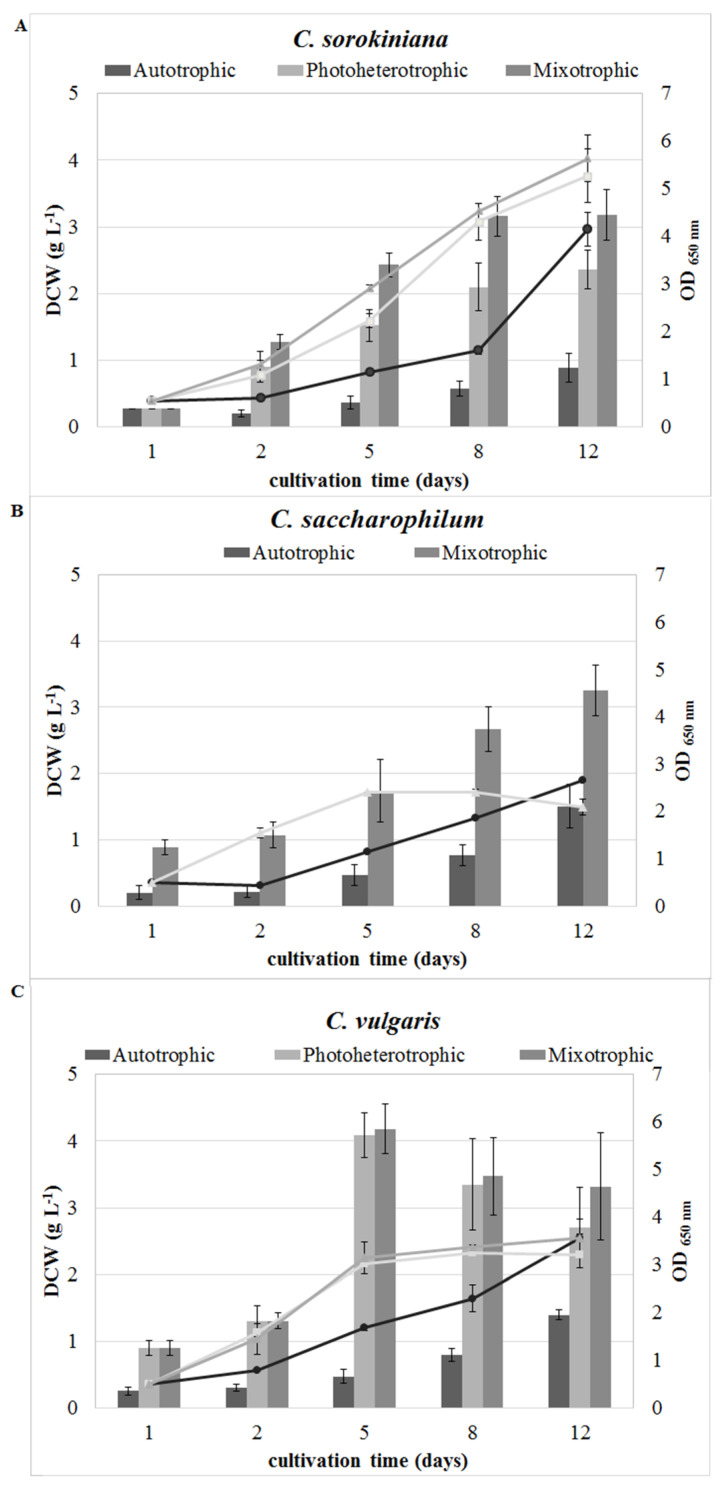
Influence of trophic modes on growth curves and dry weight of *C. sorokiniana* (**A**), *C. saccharophilum* (**B**), and *C. vulgaris* (**C**) cells (the results are represented as mean values of measurements ± SD).

The estimation of growth through OD_650_ and the dry weight of *C. sorokiniana*, *C. saccharophilum*, and *C. vulgaris* cells during cultivation in the autotrophic, photoheterotrophic, and mixotrophic modes are presented in Figure 1.

A significant influence of the trophic modes on the course of the growth curves was observed. This observation was highly similar for all the *Chlorella* species. The autotrophic growth curves of *C. sorokiniana*, *C. saccharophilum*, and *C. vulgaris* had a linear course. In the photoheterotrophic and mixotrophic modes of *C. saccharophilum* and *C. vulgaris* growth, stationary phases were reached on the 5th day of cultivation. The *C. sorokiniana* cells grew at the same rapid pace and did not reach the stationary phase over the 12 experimental days.

In the mixotrophic culture conditions, *C. vulgaris* exhibited the highest biomass yield (3.32 g L^−1^) on day 12 of the experiment. In the photoheterotrophic *C. sorokiniana* and *C. vulgaris* cultures, the biomass yield reached 2.36 g L^−1^ and 2.70 g L^−1^, respectively. *C. sorokiniana* and *C. vulgaris* were only able to grow in the photoheterotrophic mode. Within 12 days, the autotrophic *C. saccharophilum, C. sorokiniana*, and *C. vulgaris* cells reached 1.51 g L^−1^, 0.89 g L^−1^, and 1.40 g L^−1^ of biomass yield, respectively.

Specific growth rates, biomass doubling times, and biomass productivity of *Chlorella* species grown in the different trophic mode variants are shown in Table 1.

Similarly, the growth parameters were significantly affected by the culture conditions. The maximum specific growth rates (0–5 day^−1^) were recorded in the mixotrophic cultivation mode, i.e., 0.46 day^−1^, 0.44 day^−1^, and 0.39 day^−1^ for *C. vulgaris*, *C. sorokiniana*, and *C. saccharophilum*, respectively. Among the analyzed *Chlorella* species, *C. vulgaris* cell biomass increased at the fastest rate, which was additionally evidenced by the shortest doubling time of 36.47 h.

The photoheterotrophic cultivation mode of *C. sorokiniana* was characterized by a significantly lower specific growth rate and biomass doubling time, compared to mixotrophy. The differences in the *C. vulgaris* photoheterotrophic culture were not statistically significant.

The autotrophic *C. sorokiniana* cells were characterized by the weakest growth (0.19 day^−1^, 90 h). As shown in Table 1, the type of trophy had an impact on the daily biomass productivity in the *Chlorella* species. The highest biomass productivity per day was obtained in the mixotrophic cultures of *C. vulgaris*, *C. saccharophilum,* and *C. sorokiniana*. As in the case of the other growth parameters, the lowest biomass productivity per day was recorded in the autotrophic *C. sorokiniana* culture conditions.

### 2.2. Characterization of Lipids and Carbohydrates

**Figure 2:**
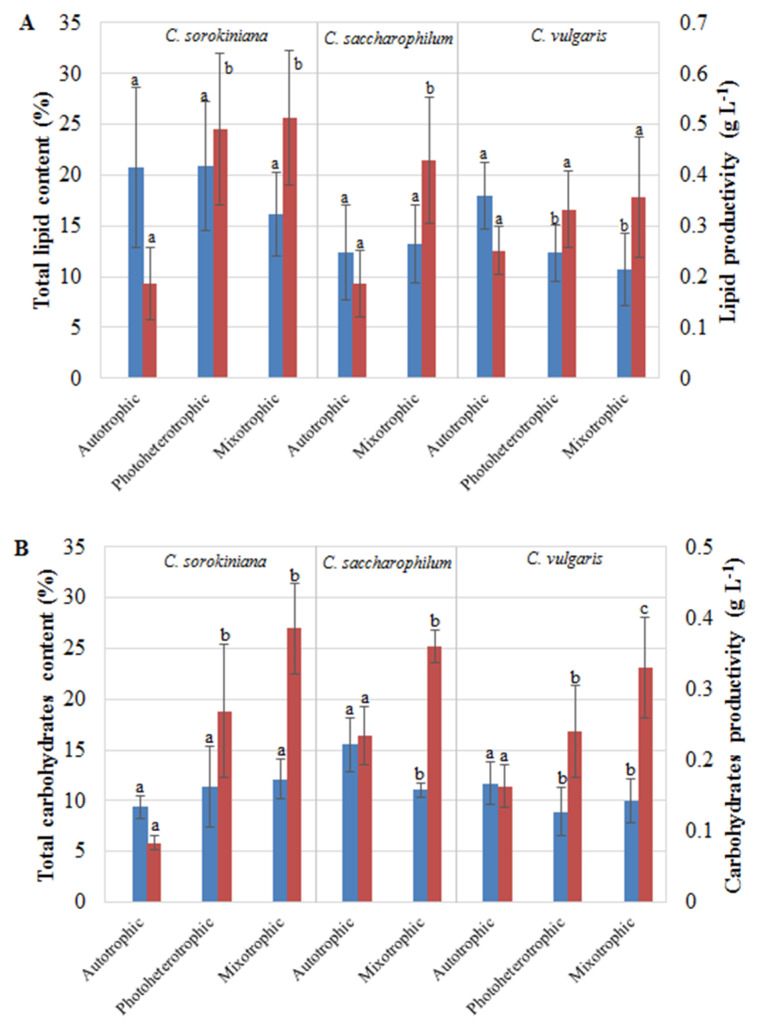
Influence of trophic modes on lipids (**A**) and carbohydrates (**B**) of *C. sorokiniana*, *C. saccharophilum*, and *C. vulgaris.* The blue columns represent the total lipid and the carbohydrate contents, and the red columns represent productivities. (the results are presented as a mean of measurements ± SD; the same letter means no significant differences; Tukey’s honestly significant difference test at *p* < 0.05).

The effects of the autotrophic, heterotrophic, and mixotrophic conditions on the lipid content and productivity in the three *Chlorella* species are presented in Figure 2A.

The statistical analysis showed that the trophic mode had a significant impact on lipid content only in the *C. vulgaris* cells. There was a statistically significant decrease in the content of total lipids from 17.99% (autotrophy) to 12.33% (photoheterotrophy) and 10.75% (mixotrophy). In turn, the greatest accumulation was observed in the autotrophic and photoheterotrophic *C. sorokiniana* cells, which reached 20.81% and 20.84%, respectively. In contrast, the nutritional strategy type significantly increased the lipid productivity in *C. sorokiniana* and *C. saccharophilum.* Maximum lipid productivity of 0.51 g L^−1^ was reached in the mixotrophic *C. sorokiniana* cultures.

The amount of total carbohydrates and carbohydrate productivity are summarized in Figure 2B. In all the *Chlorella* cultures, the carbohydrate contents were similar: they were relatively low and represented from 8.90% to 15.51% of dry cell weight. In the *C. saccharophilum* and *C. vulgaris* cells, the amount of total carbohydrates obtained in the autotrophic culture conditions differed significantly from those recorded in the photoheterotrophic and mixotrophic cultures. A significant decrease in the total amount of carbohydrates was observed in the mixotrophic *C. saccharophilum* and *C. vulgaris* cells and in the photoheterotrophic *C. vulgaris* cells. Similar to lipids, the maximum amount of total carbohydrates was produced autotrophically by the *C. saccharophilum* cells. The maximum carbohydrate productivity of 0.39 g L^−1^ was exhibited by the *C. sorokiniana* mixotrophic cells.

### 2.3. Characterization of Protein and Pigments

**Figure 3:**
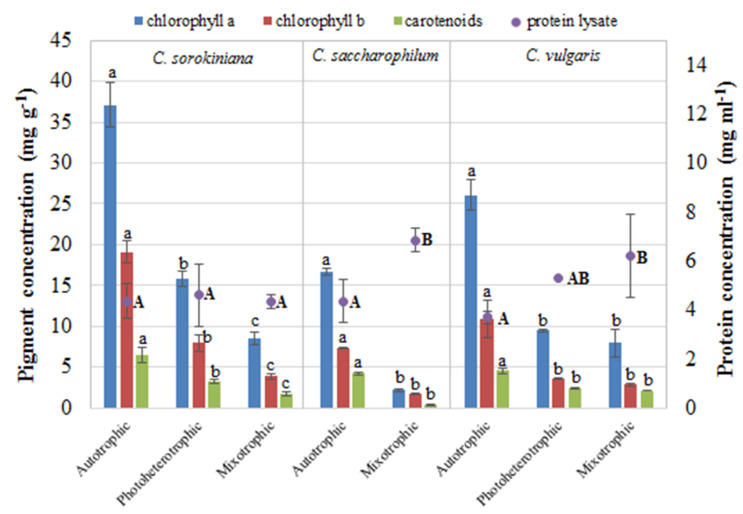
Influence of trophic modes on chlorophyll a (blue columns), chlorophyll b (red columns), carotenoids (green columns), and protein concentrations (purple dots) in *C. sorokiniana*, *C. saccharophilum*, and *C. vulgaris* (the results are presented as a mean of measurements ± SD; the same letter means no significant differences; Tukey’s honestly significant difference test at *p* < 0.05).

The influence of the trophic modes on the concentration of chlorophyll a, chlorophyll b, carotenoids, and protein in the lysate is shown in Figure 3.

The photoheterotrophic and mixotrophic modes had a significant impact on the content of all photosynthetic pigments in all the *Chlorella* species. The highest contents of chlorophyll a, chlorophyll b, and carotenoids were recorded in the autotrophic *C. sorokiniana* cells. In contrast, the photoheterotrophic and, especially, mixotrophic modes caused a significant decrease in all photosynthetic pigments. The 12-day culture of *C. sorokiniana* was characterized by high optical density, compared to the other *Chlorella* species with similar cell dry weight (Figure 2). This phenomenon can be explained by the highest content of photosynthetic pigments in *C. sorokiniana* cells after 12 days of culturing.

The protein concentration in the lysates from the *Chlorella* cell culture was also significantly dependent on the cultivation mode. The highest content of protein, i.e., 6.85 and 6.2 mg mL^−1^, was obtained from *C. saccharophilum* and *C. vulgaris* cells derived from the mixotrophic cultures, respectively. The culture conditions did not affect the protein content in the *C. sorokiniana* cells.

### 2.4. Electrophoretic Protein Profiles

**Figure 4:**
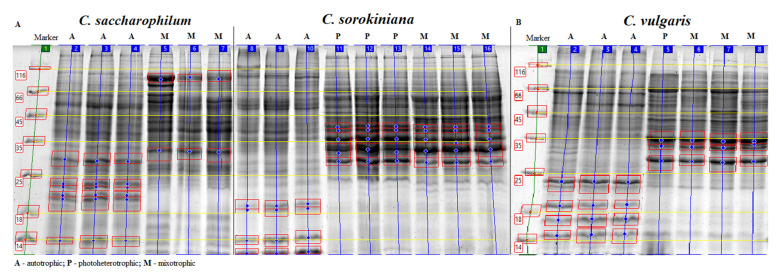
Electropherogram of proteins extracted from *C. saccharophilum*, *C. sorokiniana* (**A**), and *C. vulgaris* (**B**) cells. (**A**): Line 1 mass standard, Lines 2–4 protein lysate from autotrophically cultivated *C. saccharophila* cells, Lines 5–7 protein lysate from mixotrophically cultivated *C. saccharophilum* cells, Lines 8–10 protein lysate from autotrophically cultivated *C. sorokiniana* cells, Lines 11–13 protein lysate from photoheterotrophically cultivated *C. sorokiniana* cells, Lines 14–16 protein lysate from mixotrophically cultivated *C. sorokiniana* cells. (**B**): Line 1 mass standard, Lines 2–4 protein lysate from autotrophically cultivated *C. vulgaris* cells, Line 5 protein lysate from photoheterotrophically cultivated *C. vulgaris* cells, Lines 6–8 protein lysate from mixotrophically cultivated *C. vulgaris* cells. The mass standards are marked with the yellow line. In the figure, areas with differentially expressed protein are marked in red.

SDS-PAGE gel electrophoresis was carried out to elucidate the molecular mechanisms taking place in cells in photoheterotrophic and mixotrophic conditions. The qualitative analysis of *C. sorokiniana*, *C. saccharophilum*, and *C. vulgaris* proteins separated by SDS-PAGE and stained by Coomassie Brilliant Blue R-250 is presented in Figure 4.

The electropherogram of proteins extracted from all *Chlorella* species indicated that there were clear quantitative and qualitative differences between the protein profiles; in particular, they resulted from the culture conditions rather than the use of different species. Protein fractions ranging in molecular weights from 14 to 116 kDa were observed. Some bands were common in all the samples. Furthermore, some bands were specific for autotrophic or photoheterotrophic and mixotrophic growth. Generally, there were distinct differential protein expression patterns between the autotrophic, photoheterotrophic, and mixotrophic conditions. Differences between the autotrophic, photoheterotrophic, and mixotrophic protein profiles were observed most clearly in molecular weight ranges from 10 to 25 kDa. In the C. *saccharophilum* cells, proteins with molecular weights of about 14, 19, 20, 22, and 23 kDa were most prominent in the protein lysate from the autotrophic culture conditions. Similarly, proteins with molecular weights of ca. 11, 14, 18, and 19 kDa and 14, 17, 19, and 23 kDa were found in the autotrophic *C. sorokiniana* and *C. vulgaris* cells, respectively. One of the characteristics of the electropherogram from photoheterotrophic and mixotrophic cultures is the absence of several bands in the range from 10 to 25 kDa. Protein fractions from 10 to 25 kDa were very poorly detectable in the photoheterotrophic and mixotrophic cultures of all the investigated species. Compared to the autotrophic cell lines, the photoheterotrophic and mixotrophic *Chlorella* cells exhibited higher total protein expression levels, especially in the range from 28 kDa to 116 kDa. In addition, 28–34 kDa bands with higher density were found in the photoheterotrophic and mixotrophic cultures of *C. sorokiniana* and *C. vulgaris*. In the *C. sorokiniana* cells, 39 kDa to 40 kDa bands with an elevated concentration were observed, whereas only one 32 kDa band was present in *C. saccharophilum* cells compared with the autotrophic culture conditions in each case. Some unique bands, e.g., 66 and 89 kDa, exhibited higher density in the photoheterotrophic and mixotrophic cultures of *C. vulgaris* and *C. saccharophilum*, respectively. Not all proteins could be isolated, as the bands did not have clear boundaries and were densely arranged. The regions marked on the electropherogram may be potential markers distinguishing samples from the autotrophic cultures and those grown in the presence of organic carbon (photoheterotrophic and mixotrophic).

### 2.5. Up-Regulated Protein in Photoheterotrophic and Mixotrophic Chlorella Cultures

**Figure 5:**
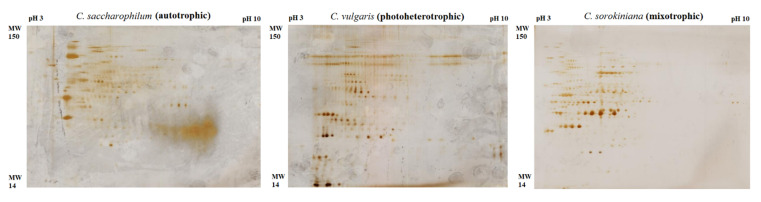
Representative 2-DE silver staining protein maps of *C. sorokiniana*, *C. saccharophilum*, and *C. vulgaris* cells.

Two-dimensional gel electrophoresis with silver staining of the samples was applied to achieve better resolution of the protein fractions. The representative 2-DE protein maps of *Chlorella* species are presented in Figure 5 (other 2-DE protein maps in Supplementary Materials).

The combination of two-dimensional gel electrophoresis (2-DE) and mass spectrometry helped to identify proteins that were up-regulated in the photoheterotrophic and mixotrophic *C. sorokiniana*, *C. saccharophilum*, and *C. vulgaris* cells. As shown by the detailed analysis, 8 proteins in the photoheterotrophy and mixotrophy variants showed a significantly higher expression level, compared to the autotrophic mode. The description of the identified up-regulated proteins, i.e., protein names, species and type of cultivation, theoretical molecular weight and observed molecular weight, pI, fold change, and molecular function and localization, is summarized in Table 2.

Glucose-6-phosphate 1-dehydrogenase, representing oxidoreductases, was the most up-regulated protein in the *C. saccharophilum* cells growing in the mixotrophic cultures. The protein is involved in carbohydrate metabolism and takes part in the oxidative phase of the pentose phosphate pathway. ATP synthase (subunit beta) involved in energy metabolism was overexpressed in the cells of *C. sorokiniana* growing in the mixotrophic cultures. The function of this protein consists in coupling the electrochemical proton gradient across the biological membrane with the synthesis of ATP, a universal biologically useful energy carrier, from ADP and phosphate. Chloroplast light-harvesting complex II was another protein overexpressed in the *C. sorokiniana* cells growing in the mixotrophic cultures and involved in protein synthesis. This protein belongs to the light-harvesting chlorophyll a/b-binding (LHC) protein family. The light-harvesting complex (LHC) functions as a light receptor. It captures and delivers excitation energy to photosystems with which it is closely associated. Three proteins of stress response were identified in the *C. saccharophilum* cells growing in the mixotrophic cultures and in the *C. vulgaris* cells from the photoheterotrophic culture variant. The results showed an increased level of luminal binding protein in the mixotrophic culture of the *C. saccharophilum* cells. Also, Hsp 70 and Hsp 90 accumulated in the *C. vulgaris* cells growing in the photoheterotrophic culture conditions. Heat shock proteins are typical representatives of the chaperone group. The photoheterotrophic *C. vulgaris* cells accumulated proteins involved in intracellular movements and growth, i.e., dynein and α-tubulin, which were up-regulated as well.

## 3. Discussion

Carbon is a crucial component for microalgae, as it plays an important role in cell nutrition. It influences not only the process of growth and cell divisions but also photosynthesis, cell structure, and biochemical composition. Sugars produced in cells during carbon metabolism play a key role in the regulation of metabolism and various life processes [16]. In the present study, the mixotrophic conditions with supplementation of the agro-industrial by-product promoted the growth of all the *Chlorella* species and accelerated the onset of the exponential growth phase, compared with the autotrophic culture. This effect was confirmed by the specific growth rate, biomass doubling time, and algal biomass concentrations. The mixotrophic *Chlorella* species showed the highest biomass yields and productivities as well as higher specific growth rates with the shortest biomass doubling times. Among all the *Chlorella* species, the mixotrophic *C. vulgaris* cells achieved the maximum results. According to relevant literature reports, mixotrophic cultures of *C. vulgaris* and *C. sorokiniana* with supplementation of agro-industrial residues and wastes were found to enhance biomass production [17,18]. As shown in our previous studies, mixotrophic cultivation promotes the growth and biomass yield of unicellular Chlorophyta green algae *Parachlorella kessleri*, and *Tetradesmus obliquus* [5,19]. The mixotrophic cultivation of *C. saccharophilum* has not been investigated to date. Our research indicated that *C. saccharophilum* utilized molasses as a carbon substrate for the production of biomass with a simultaneous supply of CO_2_ from air.

The effects of variations in nutritional strategies on the biochemical composition of *Chlorella* species were evaluated in the present study. Generally, as demonstrated by our research results and literature data, a decline in the content of total lipids and carbohydrates with an increase in the protein content was noted in *Chlorella* cells. Additionally, the total lipid content was higher in autotrophic culture conditions [20]. The changing conditions from autotrophy to photoheterotrophy and mixotrophy induced no statistically significant differences in the lipid content of *C. sorokiniana* and *C. saccharophilum* cells. In optimal growth conditions, microalgae accumulate only a limited amount of lipids and carbohydrates [21]. In the presence of stressors, microalgae synthesize lipids and carbohydrates, which are part of the protective mechanism in the cell. In order to protect microalgal cells from the excessive reducing equivalents produced under stress, lipids were accumulated in large quantities due to the greater demands of NADPH [22]. The mixotrophic conditions used in the present study seemed to be optimal and beneficial for growth but did not favor accumulation of lipids and carbohydrates. There are several reports in the literature on the use of agro-industrial wastes and residues in mixotrophic cultures of *C. vulgaris*; however, they were focused on the synthesis of lipids and fatty acids or biofuel production. The reports show the contents of lipids and carbohydrates in the range of 7.7–34.4% and 5.5–28%, respectively [17,23,24,25,26]. As reported by Leon-Vaz et al. [18], *C. sorokiniana* fed-batch mixotrophic cultivation with agro-industrial waste (wine lees) enhanced only lipid productivity. *C. saccharophilum* has never been investigated in this respect.

The newest literature review [12] shows that there are only several reports of assessment of microalgal proteins in mixotrophic conditions. Additionally, cultures with enhanced protein production are poorly documented and analyzed, and the results of research on the biochemical composition of algal cells in mixotrophic conditions vary. Some researchers have reported an increase in protein yields in cells cultured in media supplemented with organic carbon sources, in comparison to those cultivated in autotrophic conditions. Besides the nutritional strategy, the nitrogen content is directly involved in the synthesis and accumulation of cellular proteins, as the carbon/nitrogen (C/N) ratio controls the switch between protein and lipid synthesis. In our research, the protein concentration in the *Chlorella* cells, especially in *C. saccharophilum* and *C. vulgaris*, was proved to be influenced by the mixotrophic cultivation mode. The highest protein concentration in the cell lysate was obtained for *C. saccharophilum* cultured mixotrophically. An additional nitrogen source in the culture medium promotes the synthesis and accumulation of protein [5,27,28,29]. The protein content in the biomass obtained in molasses-supplemented cultivation medium is higher compared to other commercial media [26]. The pigment content was affected mainly by the trophic conditions [30]. The results of our investigations confirmed the finding that the type of nutrition influenced the pigment content in the three *Chlorella* species. In the mixotrophic growth conditions, the cells of all the *Chlorella* species were characterized by the lowest content of chlorophyll a, chlorophyll b, and carotenoids, compared to the autotrophic cultures. In the literature, *C*. *sorokiniana* cells were also characterized by reduced chlorophyll content in mixotrophic cultures, compared to autotrophy, but with essentially unaffected photosynthetic properties [31].

The electrophoretic analysis clearly showed differential protein expression patterns between the autotrophic, photoheterotrophic, and mixotrophic growth conditions. The literature does not provide reports on electrophoretic separations or comparative proteomic data on cells growing in autotrophic, photoheterotrophic, and mixotrophic growth conditions, especially *C. sorokiniana* and *C. saccharophilum*; hence, there is no basis for discussion of the present results.

However, protein profiles from Interval 4–36 kDa in green freshwater and blue-green algae mainly comprise polypeptides originating from photosystems I and II, and the proteins from Interval 8–18 kDa may be polypeptides from thylakoid membranes, LHC proteins, or phytochelatins [32]. The band of the 14 kDa protein in the autotrophic culture of all the *Chlorella* species may be related to the Ribulose bisphosphate small subunit [33]. Other authors confirmed the presence of this 15 kDa RubiSCo small subunit. In cells of *Chlorella vulgaris* in nitrate-replete and -deplete conditions [34]. In a heterotrophic-Na^+^ strategy in *Chlorella vulgaris* cultivation, 13.12 and 13.80 kDa proteins were classified as chloroplast small heat shock protein and diacylglycerol kinase participating in lipid synthesis [35]. Tejano et al. [6] identified a protein with a molecular weight of 13.58 kDa as a 50 S ribosomal protein. As shown by Sharma et al. [36], 17–21 kDa protein bands may be related to early light-induced proteins (ELIPs). The 20 kDa and 22 kDa proteins detected in the present study may be a photosystem I subunit chloroplast precursor and glyceraldehyde-3-phosphate dehydrogenase type I, respectively [34]. According to literature data, proteins involved in photosynthesis are LHC I protein with a weight of 27.4 kDa, 30.15 kDa [37], and 23 kDa [34] and LHC II protein with a weight of 26.02, 27.46 [37], and 27 kDa [34]. The 39 and 40 kDa proteins clearly visible in the electrophoresis gel may be related to magnesium chelatase CHLII and protochlorophyllide reductase POR, respectively. Nevertheless, the image of the electrophoretic separation cannot be the basis for identification of individual proteins. In turn, electrophoretic separation helps to identify differences in protein profiles and allows preliminary identification of regions that may be protein markers distinguishing *Chlorella* cells cultivated in autotrophic conditions from cells grown in the presence of OCSs. Proteomic methods, i.e., two-dimensional electrophoresis and mass spectrometry, can facilitate identification of individual proteins and detection of differences in their expression.

The proteomic analysis of the *Chlorella* cells showed an intensified synthesis of proteins in the photoheterotrophic and mixotrophic cells, which are involved in the photosynthesis and protein production (chloroplast light-harvesting complex II), energy metabolism processes (ATP synthase subunit beta), carbohydrate metabolism processes (glucose-6-phosphate 1 DH precursor), stress responses (luminal binding protein, Hsp70, Hsp90), and intracellular movements (dynein and α-tubulin).

Increased expression of the chloroplast light-harvesting complex II protein was observed in the mixotrophic *C. sorokiniana* cells. As reported by Arora et al. [17], increased levels of photosystem II (PS II) proteins in mixotrophic cultures are associated with cellular quenching of excess electrons. Supplementation of the culture medium with organic carbon decreases the dependence of cells on photosynthesis and causes up-regulation in the mitochondrial electron transport chain. These data suggest that the higher growth rates in the mixotrophic cultivation mode were a result of cellular energy generation by mitochondria. Additionally, the authors observed an increasing level of mitochondrial ATP synthase subunits α and β as an effect of sugar addition. Increased content of nitrogen in the medium is another factor inducing an increase in the concentration of PSII proteins [34]. As indicated by previous research results [5], due to its content of nitrite, nitrate, glutamic acid, and aspartic acid, beet molasses is a source of additional nitrogen in the medium and may increase the expression of proteins involved in the photosynthesis process in mixotrophic conditions. The mixotrophic mode caused changes in carbohydrate metabolism through an increase in glucose-6-phosphate 1 DH levels in the *C. saccharophilum* cells. It has been reported that supplementation of culture media with OCSs under mixotrophy enhances glycolysis [38,39]. In our research, the photoheterotrophic and mixotrophic modes of cultivation of *C. saccharophilum* and *C. vulgaris* induced the synthesis of heat shock proteins Hsp70 and Hsp90 and luminal binding protein. The production of chaperones called heat shock proteins is a strategy to maintain cellular homeostasis in response to oxidative stress [40]. Similar increases in the level of HSP 70 expression have been reported by Arora et al. [17]. Supplementation of organic carbon in the mixotrophic cultivation mode interferes with the photosynthesis process by forming such ROS as singlet oxygen, superoxide, and hydrogen peroxide [41].

Our results also showed up-regulation of proteins involved in intracellular movements. It is supposed that accumulation of these intracellular movement-related proteins, e.g., dynein and α-tubulin, might be related to intensive cell division in photoheterotrophic *C. vulgaris* cultures.

## 4. Materials and Methods

### 4.1. Algal Strains and Culture Conditions

**Figure 6:**
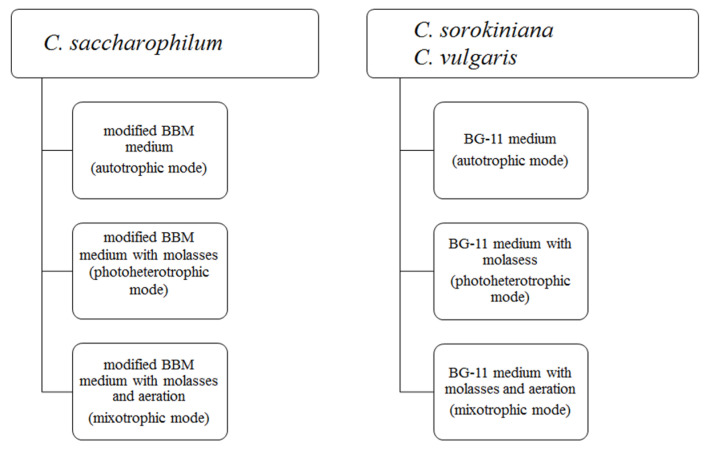
Scheme of the experiment.

Three *Chlorella* strains, *Chlorella sorokiniana* (strain No. 259), *Chloroidium saccharophilum* (formerly *Chlorella saccharophila*) (Strain No. 258), and *Chlorella vulgaris* (Strain No. 788), were obtained from the Culture Collection of Autotrophic Organisms (CCALA) in Prague. All *Chlorella* cultures were carried out in 1 L Erlenmeyer flasks containing 400 mL of medium on laboratory shakers (90 rpm) at constant temperature (20 ± 1 °C) and lighting (photosynthetically active radiation of 80 µmol photons m^−2^ s^−1^). The culture media were adjusted to the basic nutritional requirements of algal species. *C. sorokiniana* and *C. vulgaris* were cultured on BG-11 medium (Cyanobacteria BG-11 Freshwater Solution, Sigma Aldrich, Lublin, Poland) while BBM medium [42] enriched with soil extract (0.1% *w*/*v*) was used for *C. saccharophilum* cultivation. In the photoheterotrophic and mixotrophic *Chlorella* cultures, beet molasses was used for medium supplementation. Beet molasses was obtained from a local sugar refinery and was used for preparation of a 1% culture solution (*w*/*v*). Before use, molasses was rinsed with demineralized water twice and sterilized. The autotrophic and mixotrophic cultures of *Chlorella* strains were continuously aerated with sterile air. The scheme of proper batch cultures is presented in Figure 6.

### 4.2. Growth Characteristics

*Chlorella* growth was monitored by measurements of the optical density at 650 nm of the cell cultures using a Cary 300 Biomelt spectrophotometer. Additionally, the dry cell weight was determined in cell suspension vacuum filtered through a pre-weighed glass microfiber Whatman GF/C filter and dried to a constant weight at 90 °C overnight. Dry cell weight (DCW) was expressed in g L^−1^. Biomass productivity was expressed in mg L^−1^ day ^−1^. The specific growth rate (0–5 days) (µ) was calculated on the basis of the optical density using the following formula (Equation (1)):

µ (d^−1^) = ln (N_2_/N_1_)/(T_2_ − T_1_) (1)

where N_1_ and N_2_ are the optical density at T_1_ and T_2_, respectively.

In turn, the doubling time (0–5) (T_d_) was calculated with the formula below (Equation (2)).

T_d_ = (ln 2/µ) 24 (2)

### 4.3. Determination of Lipids and Carbohydrates

*Chlorella* biomass was harvested by centrifugation at 6500 rpm (Rotanta 460, Hettich^®^, Lublin, Poland) after 12 days of cultivation. The collected cells were washed twice with demineralized water to remove the medium residues and centrifuged again. The cells were again re-suspended in demineralized water to determine the dry cell weight used for calculation of the percentage of lipid and carbohydrate dry weight. A modified Bligh and Dyer method was applied for isolation of total lipids from *Chlorella* cells using methanol and chloroform [43,44]. Prior to the extraction, ultrasound was applied for cell disruption (Vibra cell 500, Sonics, Lublin, Poland). The total lipid content was determined gravimetrically and expressed in % (*m*/*m*).

The technique used for colorimetric estimation of total simple sugars was the anthrone commonly used method described by Trevelyan et al. [45]. The total carbohydrate content was determined from the glucose standard curve equation and expressed in % (*m*/*m*).

The percentage contents of cellular components, i.e., lipid and carbohydrate productivities, were expressed in g L^−1^.

### 4.4. Measurement of Photosynthetic Pigments

The content of chlorophyll a, chlorophyll b, and total carotenoids was determined spectrophotometrically according to the procedure described by Pawlik-Skowrońska et al. [46] and Welburn et al. [47]. The photosynthetic pigments were extracted using DMSO at 65° for 1 h. The total content of pigments was calculated using the following equations (Equations (3)–(5)):

Chl *a* = {[12.19 (A_665_ − A_730_)] − [(3.45 (A_649_ − A_730_)]} (V_DMSO_/V_s_) (3)

Chl *b* = {[(21.99 (A_649_ − A_730_)] − [(5.32 (A_665_ − A_730_)]}(V_DMSO_/V_s_) (4)

Carotenoids = [1000 (A_480_ − A_730_) − 2.14 Chl *a* − 70.16 Chl *b*]/220 (V_DMSO_/V_s_) (5)

where V_DMSO_ is the volume of DMSO used for the extraction and V_s_ is the volume of the cell suspension. The results obtained using these formulas and expressed in µg mL^−1^ were converted into mg g^−1^ DCW.

### 4.5. Protein Extraction, Determination and SDS-PAGE Electrophoresis

Proteins were isolated from *Chlorella* cells based on the protocols described by Jia et al. [48] and Cid et al. [49]. Lyophilized algal cells were used for protein extraction. The algal cell pellet was suspended in lysis buffer (60 mM Tris-HCl, pH 7.9, 1 mM EDTA, and 14 mM β-mercaptoethanol) in a proportion of 1 g of the pellet to *5* mL of the lysis buffer. Three freeze–thaw cycles and sonication were applied to disrupt the cells. The supernatant was decanted after centrifugation at 15,000× *g* for 45 min at 4 °C. Next, the proteins in the supernatant were precipitated at −20 °C overnight by adding 10% TCA in 80% acetone. Proteins were separated by centrifugation at 15,000× *g* for 30 min, washed twice with cold acetone, and re-suspended in a buffer containing 50 mM Tris-HCl pH 8.0, 2 M thiourea, 7 M urea, 1 mM DTT, and 2% CHAPS. The proteins were quantified with the Bradford method [50], which is based on spectrophotometric measurements of the absorbance of a complex mixture consisting of the Bradford reagent and the cell supernatant. A total amount of the protein-containing sample, 30 µL, was mixed with 900 µL of Bradford reagent and measured at the wavelength of 595 nm using a UV-Vis spectrophotometer (Pharmacia Biotech, Lublin, Poland). This protein determination method involves the binding of Coomassie Brilliant Blue G-250 to protein in the lysate. The readings obtained in the absorbance unit were converted into the protein concentration using the calibration curve obtained using BSA as standard in a range of 0–1 mg L^−1^. SDS polyacrylamide gel electrophoresis was used for separation and characterization of proteins. SDS facilitates electrophoretic separation of proteins according to their molecular weight, as proteins are dissociated and denatured in the presence of SDS. The amount of protein placed in Eppendorf tubes ensured a 50 µg/mL protein concentration in the gel. Water was added into the tubes, and an identical amount of loading buffer was added to each sample. The samples were concentrated by incubation at 100 °C in a thermoblock for 5 min. The separation was carried out in a discontinuous polyacrylamide gel system with 12.5% of separation gel and 6% of thickening gel. The unstained protein MW Marker (Thermo Scientific, Lublin, Poland) was used for the electrophoresis. The gel electrophoresis was performed mostly in triplicate for each condition. Images of the SDS-PAGE gels were used for determination of protein molecular weight with the use of Total Lab Quant analysis software ver.13.2: TL100-3JAF-G7AG-M4 developed by TotalLab Ltd. (Gosforth, UK) (www.totallab.com, accessed on 15 November 2021).

### 4.6. 2-DE Electrophoresis and MALDI/TOF Analysis

Two-dimensional gel electrophoresis (2-DE) electrophoresis was carried out as described by Jia et al. [49]. The first dimension in the separation was carried out on 18 cm long IPG strips with a pH gradient of 3 to 10 (Immobiline^®^ DryStrip gels, GE Healthcare, Lublin, Poland) in the presence of IPG Buffer using GE Healthcare Ettan^™^ IPGphor 3 according to the manufacturer’s instructions for 17 h. After the first dimension, the IEF strips were applied for the second dimension step in SDS-PAGE electrophoresis carried out on 12.5% acrylamide at a constant voltage of 150 V for 5 h. The differentially expressed proteins in the form of spots resolved by 2-DE from the preparative gels were stained with Pierce™ Silver Stain for Mass Spectrometry (Thermo Scientific) for identification of proteins. Selected proteins were manually cut and digested by trypsin. Peptides were identified by MALDI-TOF/TOF MS (Bruker UltraXtreme, Lublin, Poland). When the ratio exceeded 2.0, the differences in the protein profile were statistically significant.

### 4.7. Statistical Analysis

Each culture variant was performed in three independent biological replicates. All measurements were performed minimum in triplicate. Statistical analysis of the investigation results was carried out in the STATISTICA 13 program (StatSoft Inc., Tulsa, OK, USA). Results were tested using two-way ANOVA followed by Tukey’s honestly significant difference test at *p* < 0.05.

## 5. Conclusions

The combination of microalgal cultivation with industrial wastes, residues, and by-products makes the mixotrophic cultivation of algae more economically viable. The mixotrophic cultivation mode with beet molasses addition can obviously promote the growth of algae and change the course of their growth. It also induces changes in the biochemical composition of cells. The reaction of the analyzed *Chlorella* algae to the trophic modes was similar, but *Chlorella vulgaris* was characterized by the highest biomass yield, the fastest growth rate, the shortest biomass doubling time, and the highest daily biomass yield in the mixotrophic culture conditions. In the mixotrophic mode, the presence of an additional source of carbon and nitrogen regulates the course of metabolic processes in *Chlorella saccharophilum*, *Chlorella sorokiniana,* and *Chlorella vulgaris* cells, which results in the synthesis and accumulation of specific proteins. Already at the initial stage of determination of proteins, SDS-PAGE gel electrophoresis indicates differences in the intensity of bands in the gel related to the different content of individual proteins between the autotrophic, photoheterotrophic, and mixotrophic modes, which may be the basis for the identification and differentiation of trophic conditions in algal cultures. The use of proteomic methods facilitates the identification of proteins that are up-regulated in photoheterotrophic and mixotrophic cells, which provides broad insight into processes taking place in algal cells.

## Figures and Tables

**Table 1:** Influence of trophic modes on basic growth parameters and biomass productivity of *C. sorokiniana*, *C. saccharophilum*, and *C. vulgaris* (the results are represented as mean values of measurements ± SD; the same letter means no significant differences; Tukey’s honestly significant difference test at *p* < 0.05).

Species	*C. sorokiniana*			*C. saccharophilum*		*C. vulgaris*		
Conditions	Autotrophic	Photoheterotrophic	Mixotrophic	Autotrophic	Mixotrophic	Autotrophic	Photoheterotrophic	Mixotrophic
**Specific growth rate µ (d^−1^)**	0.19 ± 0.01 a	0.37 ± 0.02 b	0.44 ± 0.01 c	0.21 ± 0.01 a	0.39 ± 0.00 b	0.30 ± 0.01 a	0.45 ± 0.00 b	0.46 ± 0.03 b
**Doubling time (h)**	90.01 ± 5.76 a	44.63 ± 1.81 b	37.81 ± 0.57 c	81.10 ± 2.67 a	42.45 ± 0.13 b	54.98 ± 1.37 a	36.92 ± 0.21 b	36.47 ± 2.94 b
**Biomass productivity (mg L^−1^ d^−1^)**	74.38 ± 18.05 a	196.53 ± 24.07 b	264.58 ± 31.71 c	125.56 ± 27.07 a	271.04 ± 31.72 b	116.67 ± 6.80 a	225.00 ± 50.00 b	276.39 ± 67.27 b

**Table 2:** Characteristics of up-regulated proteins induced photoheterotrophically and mixotrophically in the culture of *C. sorokiniana*, *C. saccharophilum*, and *C. vulgaris*.

No.	Up-Regulated Protein	Species/Culture Conditions	Theoretical MW/MW (kDa)	pI	Fold Change	Molecular Function/Localization
1	ATP synthase subunit beta	*C. sorokiniana*/mixotrophic	42.1/39.6	5.3	3.2	Energy metabolism/chloroplast
2	Chloroplast light-harvesting complex II	*C. sorokiniana*/mixotrophic	24.0/26.1	5.0	2.9	Protein synthesis/chloroplast
3	Luminal binding protein	*C. saccharophilum*/mixotrophic	71.8/73	4.8	3.6	Stress response/ER
4	Glucose-6-phosphate 1 DH precursor	*C. saccharophilum*/mixotrophic	66.9/67	8.5	4.1	Carbohydrate metabolism/chloroplast
5	Hsp70	*C. vulgaris*/photoheterotrophic	70.9/71	5.3	2.9	Stress response/chloroplast
6	Hsp90	*C. vulgaris*/photoheterotrophic	80.7/80	4.9	2.7	Stress response/chloroplast
7	Dynein	*C. vulgaris*/photoheterotrophic	76/77	5.4	3.1	Cytoskeleton protein
8	α-tubulin	*C. vulgaris*/photoheterotrophic	49/50	5.0	2.2	Cytoskeleton protein

## Data Availability

Not applicable.

## Supplementary Materials

The following supporting information can be downloaded at: www.mdpi.com/article/10.3390/molecules27154817/s1, Figure S1: 2-DE protein map of C. saccharopilum (mixotrophic), Figure S2: 2-DE protein map of C. sorokiniana (autotrophic), Figure S3: 2-DE protein map of C. sorokiniana (photoheterotrophic), Figure S4: 2-DE protein map of C. vulgaris (autotrophic), Figure S5: 2-DE protein map of C. vulgaris (mixotrophic).

## Sample Availability

Samples of the biomass can be obtained from authors.
