# Supplementary material for: Metabolic and Proteomic Analysis of Chlorella sorokiniana, Chloroidium saccharofilum, and Chlorella vulgaris Cells Cultured in Autotrophic, Photoheterotrophic, and Mixotrophic Cultivation Modes
Source: Molecules. 2022 Jul 27;27(15):4817. doi: 10.3390/molecules27154817 (PMC9369600; doi:10.3390/molecules27154817)
Supplement: Supplementary file 1 [file molecules-27-04817-s001.zip › molecules-1784083-supplementary.pdf]

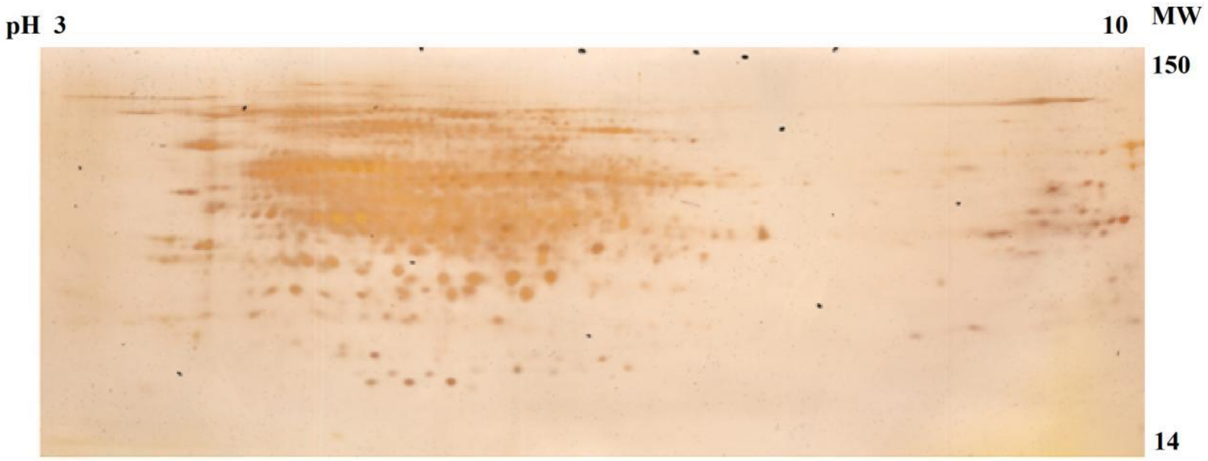

Figure S1.2-DE protein map of Chlorella saccharopilum (mixotrophic).

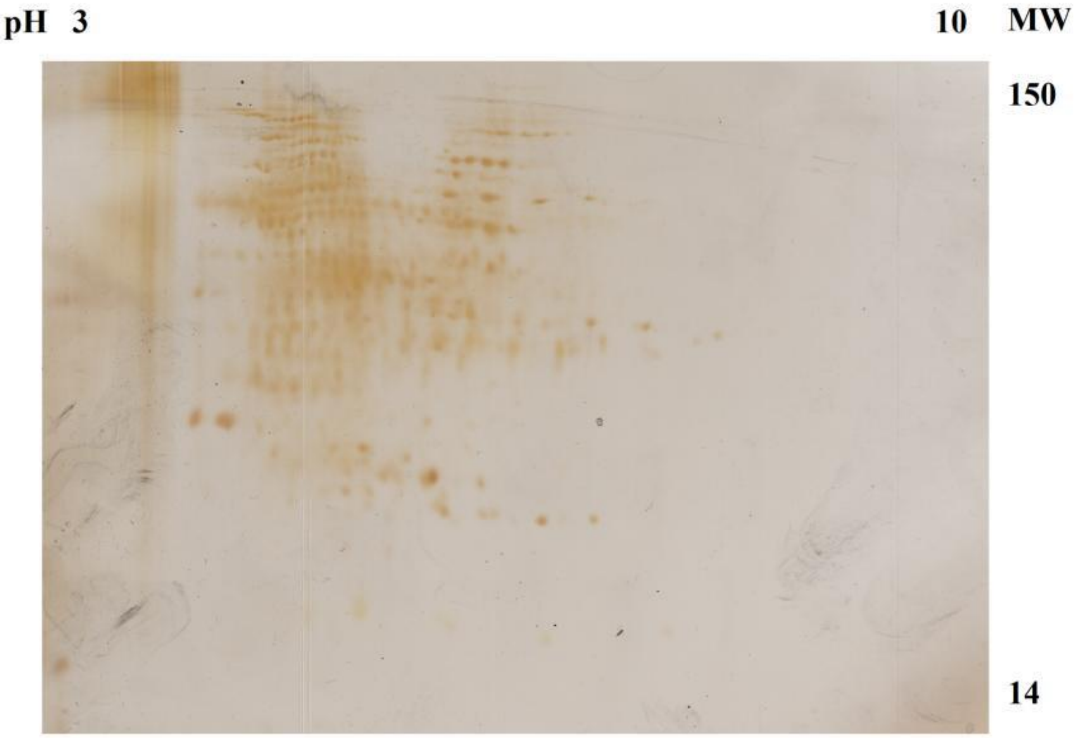

Figure S2. 2-DE protein map of Chlorella sorokiniana (autotrophic).

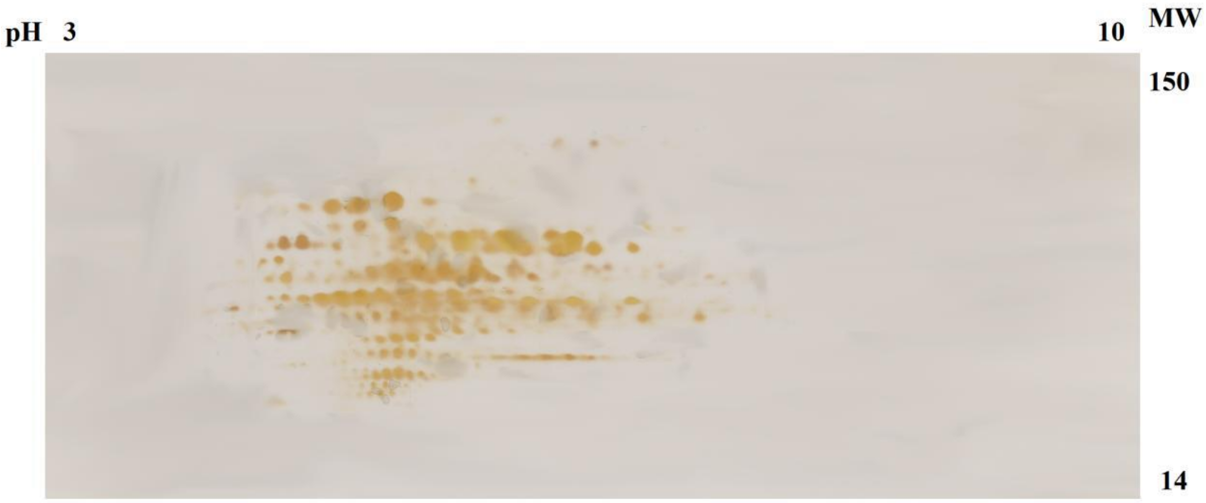

Figure S3: 2-DE protein map of Chlorella sorokiniana (photoheterotrophic).

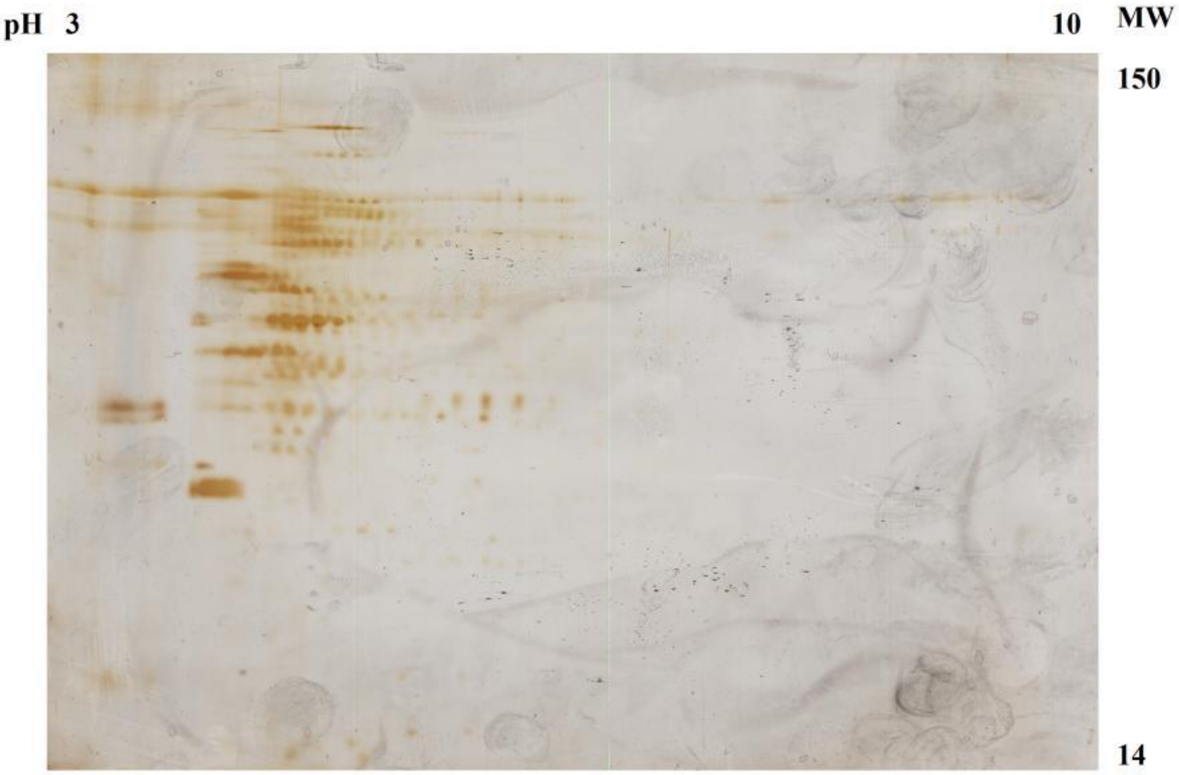

Figure S4: 2-DE protein map of Chlorella vulgaris (autotrophic).

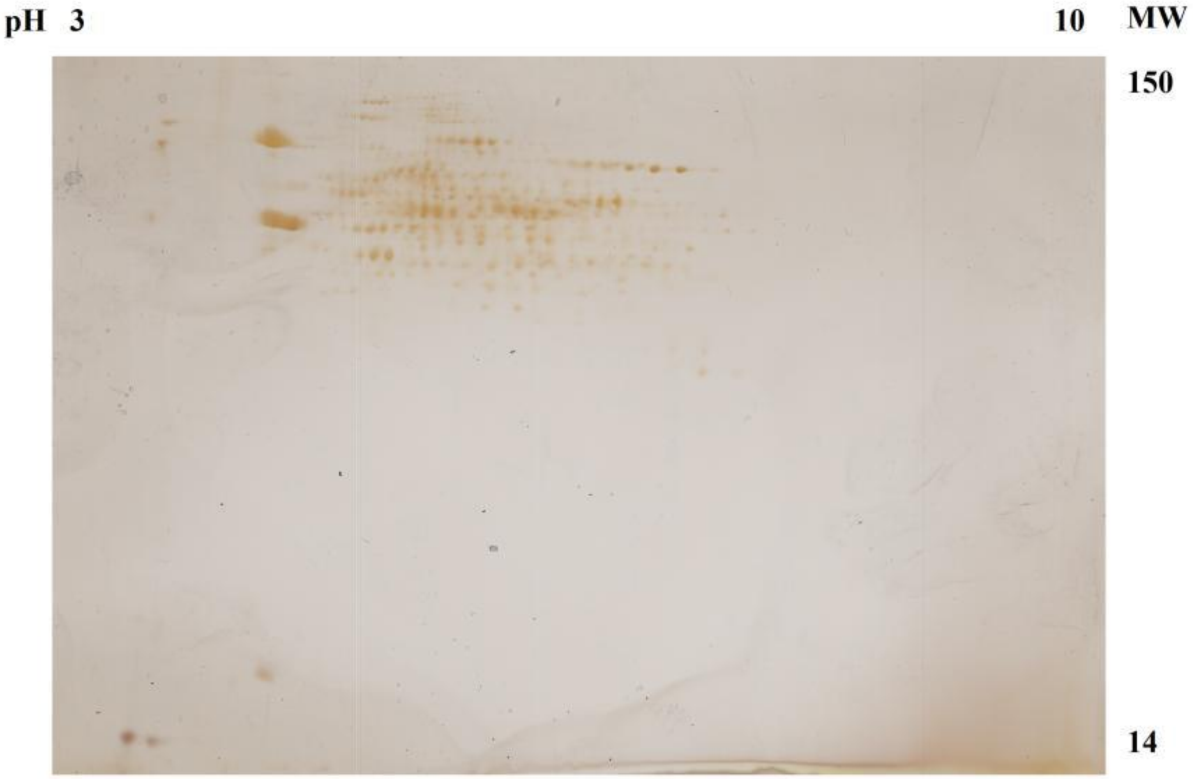

Figure S5: 2-DE protein map of Chlorella vulgaris (mixotrophic).
